# Association between social and built environments and leisure-time physical activity among Chinese older adults - a multilevel analysis

**DOI:** 10.1186/s12889-015-2684-3

**Published:** 2015-12-29

**Authors:** Junling Gao, Hua Fu, Jiang Li, Yingnan Jia

**Affiliations:** School of Public Health, Key Laboratory of Public Health Safety, Ministry of Education, Shanghai, China

## Abstract

**Background:**

Social and physical environments are not only hypothesized to influence physical activity (PA), they are also interrelated and influence each other. However, few studies have examined the relationships of PA with social and physical environments simultaneously. Accordingly, the current study aims to examine the association between physical and social attributes of neighborhood with leisure-time physical activity (LTPA) among the Chinese elders.

**Methods:**

By employing a two-stage stratified random sampling procedure, 2783 elders were identified from 47 neighborhoods in Shanghai during July and September in 2014. Social and physical attributes of neighborhood were assessed using a validated and psychometrically tested measures, and the Chinese version of the International Physical Activity Questionnaire—Long Form was used to assess LTPA. Control variables included sex, age, marital status, education level, self-rated health and chronic conditions. Multilevel logistic regression analysis was conducted to explore whether individual- and neighborhood-level social and physical attributes were associated with LTPA.

**Results:**

The overall prevalence of leisure-time active (LTA) was 46.6 %. After controlling for individual covariates, 1) compared to participants with the first quartile of social participation, the odds ratios of LTA for participants with the second, third and fourth quartile of social participation were 1.86 (95 % CI: 1.44–2.41), 2.37 (95 % CI: 1.82–3.08) and 4.27 (95 % CI: 3.27–5.58); 2) compared to participants with the first quartile of social cohesion, the odds ratios of LTA for participants with the second, third and fourth quartile of social cohesion were 1.09 (95 % CI: 1.07–1.20), 1.14 (95 % CI: 1.08–3.50) and 1.31 (95 % CI: 1.11–1.58); 3) compared to participants living in neighborhoods with the first quartile of walkability, the odds ratios of LTA for participants living in neighborhoods with the second, third and fourth quartile of walkability were 1.13 (95 % CI: 1.03–2.02), 1.73 (95 % CI: 1.12–3.21) and 1.85 (95 % CI: 1.19–3.35).

**Conclusions:**

Both social and physical attribute of neighborhood associate with LTPA among Chinese older adults. It may promote LTPA among Chinese older adults to encourage them to participate in social activities, meanwhile, building walkable and cohesive neighborhoods.

## Background

Regular participation of leisure-time physical activity (LTPA) has many benefits including postponing premature mortality [1–3], reducing the development of chronic non-communicable diseases [4–7], and improving quality of life [8–10]. LTPA is particularly relevant for elders, as they tend to have significantly more leisure time available than people in younger age cohorts [4]. Furthermore, LTPA may also provide the best opportunity to intervene compared with occupational and household physical activity [11]. Having the largest and most rapidly growing ageing population in the world [12], China is undergoing a rapid transition from a rural to an urban society. It is expected that more than 1 billion people will live in Chinese cities by 2050 [13]. Rapid urbanization may be associated with higher prevalence of chronic age-related diseases (e.g., diabetes) and unhealthy lifestyle (e.g., decreased physical activity levels) [14]. For example, most of the Chinese elderly did not engage in LTPA [15]. For many older adults, the neighborhood of residence is their predominant environmental context. The physical and social conditions of the neighborhood environment may be more important to older adults and particularly those who are retired or becoming frail and therefore likely to be spending increasingly more time with neighbors in their immediate neighborhood [16]. Exploring the unique effects of neighborhood attributes on elders’ LTPA could be helpful to urban planners and public health officials in their efforts to build age-friendly neighborhoods and cities.

The physical environment and social environments are the most important aspects of one’s surroundings that potentially influence LTPA participation [4, 17]. The physical environment is defined as the objective and perceived characteristics of the physical context in which people spend their time (e.g. home, neighborhood), including aspects of urban design (e.g. presence of sidewalks), traffic density and speed, distance to and design of venues for physical activity (PA) (e.g. parks), crime and safety [18]. Recently, more attention has been paid to physical environmental correlates of LTPA among elders, but there were no consistent results so far [19]. For example, neighborhood walkability is related to LTPA in the US [20–22], but is not related to LTPA in other countries [23, 24]. Although, there was no unified definition of social environment [25], which encompasses interpersonal relationships (e.g., social support and social networks), social inequalities (e.g., socioeconomic position and income inequality, racial discrimination), and neighborhood and community characteristics (e.g., social cohesion and social capital, neighborhood factors). The positive effects of social participation on health may be significant for elders because elders have more times to take part in social activities due to retirement or fewer familial constraints [26, 27]. The study conducted among people aged 50 years and over in 11 European countries (including Sweden) has shown that social participation was positively associated with self-rated health [28]. However, two studies in Sweden demonstrated that social participation was negatively associated LTPA among people aged 20–80 years [29, 30]. Social cohesion as another neighborhood determinant of health [31], is particularly relevant to elders because of its association with neighborhood social order and rates of violent crime [32, 33]. Studies have shown social cohesion is associated with wellbeing [34], depressive symptoms [35] and walking activity [17, 33].

Physical and social environments are not only hypothesized to influence health behaviors, they are also interrelated and influence each other [36, 37]. A previous study [38] found that adults living in high-walkable Irish neighborhoods reported higher levels of knowing their neighbors, political participation, trust in other people, and social participation compared to participants living in low-walkable area. Other studies have also supported the premise that pedestrian-friendly environments are related to increased social capital [39–41]. However, few studies have simultaneously examined associations of individual, physical and social environmental characteristics with physical activity [42].

Neighborhood attributes’ relation to physical activity are relatively well researched in Western countries, but remain largely underexplored in China. Some studies in China [43–52] have explored the relationship between environmental characteristics and LTPA, but most of them [47, 49–52] were conducted among Hong Kong elders. None of these studies have examined the relationship between social environment and LTPA. Very often environmental characteristics consist of individuals/units at a lower level nested within spatial units at a higher level (e.g., individuals nested within neighborhoods) [19, 25]. Environmental characteristics should be measured at the interpersonal level, ecological level, or both. Multilevel methods are specifically geared toward the statistical analysis of data that have nested structures and sources of variability at multiple levels [53]. Accordingly, in the present study we aim to examine the association between physical and social environments (both at individual- and neighborhood-levels) and LTPA among the Chinese elderly.

## Methods

### Participants and study design

**Fig. 1:**
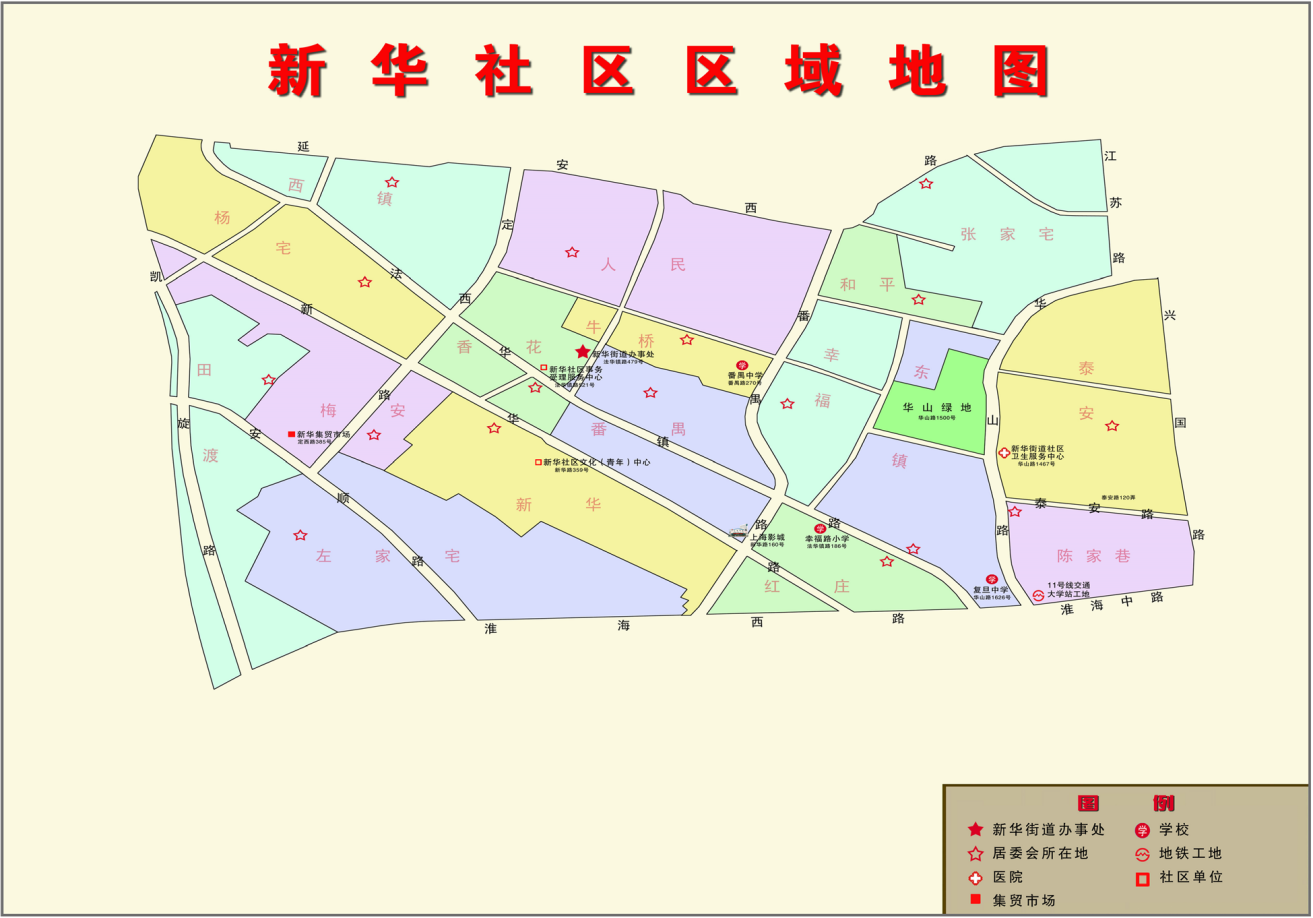
Regional areas of Xinhua Community

**Fig. 2:**
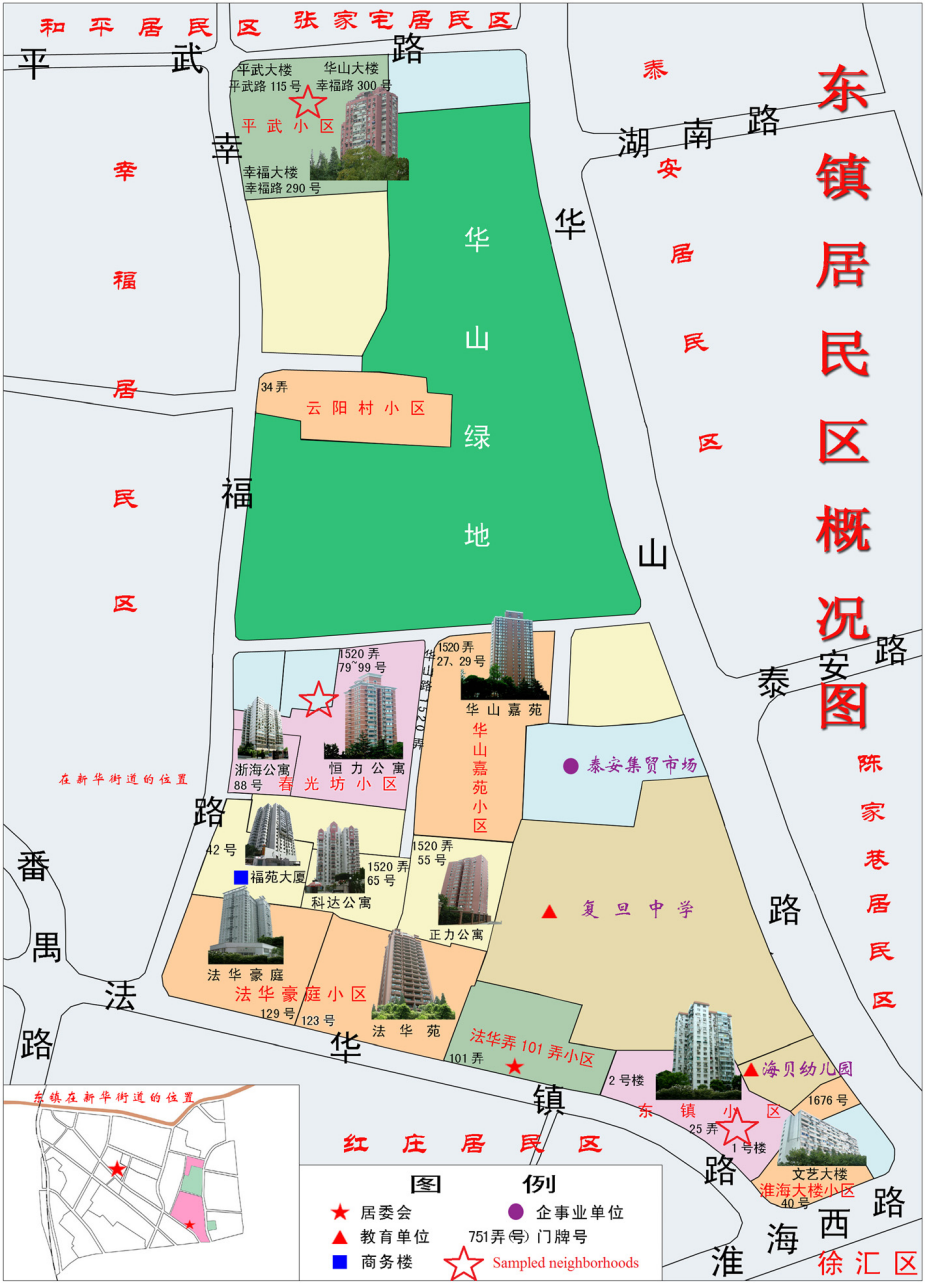
A example of sampled neighborhoods from a regional area

In China city, neighborhood was clustered administratively. Specifically, every sub-district of a city’s district administers many neighborhoods. Each of neighborhoods has a neighborhood committee to administer the dwellers of that neighborhood [54]. The current study was conducted in the Xinhua sub-district in Shanghai from July and September in 2014. This sub-district (approximately 2.2 sq. km) is located in southwest of Shanghai, consists of 17 residential areas (Fig. 1). There are 198 neighborhoods with about 78 thousand people (55) (http://www.xhjd.org/) in residential areas. 16 % of all population is aged over 65 years old. In order to explore how neighborhood’s attributes affect older adults’ participation in LTPA, two-stage sampling method was used. Firstly, we gained the maps of residential areas from Xinhua Community committee. Based on these maps, one of the authors and two workers of Xinhua Community committee selected 47 neighborhoods from 17 residential areas by purposive sampling taking diversities into account (such as accessibility to services, aesthetics, and street connectivity) (see Fig. 2 for example). Than name lists of elders aged 60 years and over without severe cognitive impairment or physical limitations were gained from neighborhood committees. Next, we used the name lists to randomly sample 120 elders from each neighborhood that has more than 120 elders; otherwise, in neighborhoods with fewer than 120 older adults, all older adults living in the neighborhood were selected.

The trained health-related workers from neighborhood committees interviewed the participants face-to-face. All the participants provided written informed consents before the interview. Totally, 2839 elders were sampled from 47 neighborhoods, however 56 elders were excluded from this analysis because of incomplete data, resulting in 2783 elders were included in the current study. The study was approved by the Institutional Review Board of the School of Public Health at Fudan University.

### Measurements

#### Leisure-time physical activity

The last 7-day weekly minutes of recreational walking, moderate, and vigorous intensity physical activity were estimated using the Chinese long form of the International Physical Activity Questionnaire [55]. The Cronbach’s alpha of items on LTPA was 0.694 for the current sample. According to the previous studies [56, 57], elders was categorized into leisure-time active (LTA) and leisure-time inactive (LTI). Leisure-time active refers to at least 150 min of leisure-time physical activity per week. This criterion is in accordance with the current recommendations for the practice of physical activity [58].

#### Attributes of neighborhood

##### Physical attributes of neighborhood

In the current study, two modules of Neighborhood Scales developed by Mujahid et al [59]. were used to assessed aesthetic quality (AQ) and walkability of neighborhood. Based on the original scale, an initial translation into Chinese was done, and then back translated into English to verify that the content of the original scale was maintained.

Module of AQ consists of 5 items: 1) There is a lot of trash and litter on the street in my neighborhood, 2) There is a lot of noise in my neighborhood. 3) In my neighborhood the buildings and homes are well-maintained, 4) The buildings and houses in my neighborhood are interesting, 5) My neighborhood is attractive. Each item ranged from 1 to 5 (1 = strongly disagree, 2 = disagree, 3 = neutral (neither agree nor disagree), 4 = agree, and 5 = strongly agree). Item 1 and item 2 were reverse-coded. The Cronbach’s alpha of original scale was 0.75 [59], which is also 0.75 for the current sample.

Module of walkability consists of 7 items: 1) My neighborhood offers many opportunities to be physically active, 2) Local sports clubs and other facilities in my neighborhood offer many opportunities to get exercise, 3) It is pleasant to walk in my neighborhood, 4) The trees in my neighborhood provide enough shade, 5) In my neighborhood it is easy to walk places, 6) I often see other people walking in my neighborhood, 7) I often see other people exercising (for example, jogging, bicycling, playing sports) in my neighborhood. Each item also ranged from 1 to 5 (1 = strongly disagree, 2 = disagree, 3 = neutral (neither agree nor disagree), 4 = agree, and 5 = strongly agree). The Cronbach’s alpha of original scale was 0.73 [59], which is 0.81 for the current sample.

##### Social attributes of neighborhood

**social participation** was assessed by asking respondents how often in the past 12 months they participated in eight different activities: 1) Visiting family or friends, 2) Recreational activities involving other people, 3) Physical and cultural activities in neighborhood, 4) Attending series of lectures in neighborhood, 5) Self-management group, mutual-help group, 6) Volunteer or charity work, 7) Activities of political organizations or associations, 8) Dinning out or shopping with others people. Each social activity ranged from 1 to 5 (1 = never, 2 = several times per year, 3 = several times per month, 4 = once per week, and 5 = two or more times per week). The Cronbach’s alpha is 0.83 for the current sample.

**Social cohesion** was assessed by the related module of Neighborhood Scales developed by Mujahid et al. [59], which consists of 4 items: 1) People around here are willing to help their neighbors, 2) People in my neighborhood generally get along with each other, 3) People in my neighborhood can be trusted, 4) People in my neighborhood share the same values. Each item also ranged from 1 to 5 (1 = strongly disagree, 2 = disagree, 3 = neutral (neither agree nor disagree), 4 = agree, and 5 = strongly agree). The Cronbach’s alpha of original scale was 0.74, [59] which is 0.88 for the current sample.

Due to association between neighborhood characteristics and individual-level characteristics [59, 60], the extent that people’s perceptions reflect reality, the averaging of responses across multiple persons within a neighborhood reduces measurement error due to individual subjectivity [59]. All attributes of neighborhood were assessed in two alternative ways: (a) Individual-level attributes, by calculating the mean score of each individual’s own assessments on the corresponding scale’s items. (b) Similar to the previous study [59], neighborhood-level attributes, by estimating mean scale score of all respondents in the same neighborhood. For analysis, both individual and neighborhood-level attribute scores were dichotomized into good versus poor for physical attributes, high versus low for social attributes by median.

#### Covariates

We selected the following variables as relevant confounders for statistical control: sex, age (5-year categories), marital status (married or cohabiting vs. other), self-reported chronic diseases (none, one, and two or more) and education (elementary school, junior high school, senior high school and university or higher.). Self-rated health was assessed by the single item: “Would you say that in general your health is excellent, very good, good, fair, or poor?” From this item, we created a dichotomous measure (0 = fair or poor; 1 = excellent, very good, or good).

### Statistical analyses

Our data had a multilevel structure comprised of elders (at first level) nested within neighborhoods (at second level). We fitted the data using multilevel logistic regression models, adjusting for both individual- and neighborhood-level variables as fixed effects and allowing for a random intercept for LTA. Adjusted odds ratios (ORs) and their 95 % confidence intervals (CIs) for LTA were obtained for both individual- and neighborhood-level attributes of neighborhood. The analyses to examine the association between attributes of neighborhood and LTA involved estimating multiple sequential models [61]. After examining the neighborhood-level variance in LTA without including any explanatory variables (null model), we examined the relationship between individual- and neighborhood-level attributes of neighborhood with LTA (model 1 and model 2, respectively). Finally, we modeled all individual- and neighborhood-level variables simultaneously (model 3). We used -2 log likelihood (-2LL) and Akaike information criterion (AIC) to compare the goodness-of-fit of each model [61]. The STATA version 13.1 was used for all analyses (StataCorp, Texas, USA).

## Results

### Descriptive results

Demographic characteristics, the corresponding prevalence of LTA, and univariate analyses are shown in Table 1. Overall, 1638 older adults were women (58.9 %), more than half of them (51.9 %) were equal or more than 70 years old. Only 16.1 % graduated from university. More than 70 % of them reported having at least one chronic disease, and 65.8 % reported poor self-rated health. The overall prevalence of LTA was 46.6 %. The prevalence was statistically significantly higher among those who were married/cohabiting (48.3 %) than among their unmarried counterparts (39.8 %). The prevalence of high LTA also differed between age groups: those aged 70 years and over had the lowest prevalence of LTA (39.8 %) whereas those aged 65–69 years had the highest prevalence of LTA (54.4 %).

**Table 1:** Comparisons the prevalence of leisure-time active (LTA) among demographic characteristics by univariate analysis

	*N* (%)	LTA *n* (%)	LTI *n* (%)	*p* value
All	2783	1297 (46.6)	1486 (53.4)	
Sex				
Men	1145 (41.1)	511 (44.6)	634 (55.4)	.081
Women	1638 (58.9)	786 (48.0)	852 (52.0)	
Age (year)				
< 65	730 (26.2)	391 (53.6)	339 (46.4)	<.001
65~	610 (21.9)	332 (54.4)	278 (45.6)	
70~	1443 (51.9)	574 (39.8)	869 (60.2)	
Education level				
Elementary school	867 (31.2)	386 (44.5)	481 (55.5)	.351
Junior high school	988 (35.5)	480 (48.6)	508 (51.4)	
Senior high school	481 (17.3)	220 (45.7)	261 (54.3)	
University	447 (16.1)	211 (47.2)	236 (52.8)	
Marital status				
Married or cohabiting	2240 (80.5)	1081 (48.3)	1159 (51.7)	<.001
Other	543 (19.5)	216 (39.8)	327 (60.2)	
Self-rated health				
Poor	1830 (65.8)	843 (46.1)	987 (53.9)	.430
Good	953 (34.2)	454 (47.6)	499 (52.4)	
Chronic diseases				.161
None	649 (23.3)	309 (47.6)	340 (52.4)	
One	1119 (40.2)	539 (48.2)	580 (51.8)	
Two or more	1015 (36.5)	449 (44.2)	566 (55.8)	

*LTA* leisure-time active, *LTI* leisure-time inactive

### Univariate analysis of attributes of neighborhood and LTA

Table 2 illustrated that the prevalence of LTA significantly ascended in conjunction with greater individual perceptions of AQ, walkability and social participation. For example, the prevalence of LTA among participants who perceived their neighborhood AQ in the first (lowest), second, third and fourth were 41.0, 46.1, 46.9 and 51.1 %, respectively. The prevalence of LTA were different among participants in different quartiles of individual perceptions of social cohesion (*p* = 0.008). Specifically, participants who perceived their neighborhoods in the third quartile of social cohesion have the highest prevalence of LTA (50.4 %).

**Table 2:** Comparisons of LTA among individual perceptions of neighborhood characteristics by univariate analysis

	*N* (%)	LTA *n* (%)	LTI *n* (%)	*p* value
Physical characteristics				
Aesthetic quality				
1^st^ quartile	630 (22.6)	258 (41.0)	372 (59.1)	.002
2^nd^ quartile	664 (23.9)	306 (46.1)	358 (53.9)	
3^rd^ quartile	659 (23.7)	309 (46.9)	350 (53.1)	
4^th^ quartile	830 (29.8)	424 (51.1)	406 (48.9)	
Walking environment				
1^st^ quartile	681 (24.5)	274 (40.2)	407 (59.8)	<.001
2^nd^ quartile	688 (24.7)	289 (42.0)	399 (58.0)	
3^rd^ quartile	709 (25.5)	341 (48.1)	368 (51.9)	
4^th^ quartile	705 (25.3)	393 (55.7)	312 (44.3)	
Social characteristics				
Social cohesion				
1^st^ quartile	688 (24.7)	290 (42.2)	398 (57.9)	.008
2^nd^ quartile	495 (17.8)	218 (44.0)	277 (56.0)	
3^rd^ quartile	494 (17.8)	249 (50.4)	245 (49.6)	
4^th^ quartile	1106 (39.7)	540 (48.8)	566 (51.2)	
Social participation				
1^st^ quartile	560 (20.1)	154 (27.5)	406 (72.5)	<.001
2^nd^ quartile	760 (27.3)	313 (41.2)	447 (58.8)	
3^rd^ quartile	677 (24.3)	329 (48.6)	348 (51.4)	
4^th^ quartile	786 (28.2)	501 (63.7)	285 (36.3)	

*LTA* leisure-time active, *LTI* leisure-time inactive

### Multilevel analyses of the relationship attributes of neighborhood and LTA

The multilevel modeling results are shown in Table 3. The null model indicated that there was a statistical significant variation in LTA across neighborhoods (χ^2^ (1) = 153.38, *p* < 0.001); the interclass correlation coefficient (ICC) was 0.125, indicating that 12.5 % of variance of the prevalence of LTA was explained by a random effect for neighborhoods. Without controlling for individual covariates, **model 1** indicated there were positive association between LTA with individual-level social cohesion, individual-level social participation and individual-level walkability. For example, compared to participants who perceived their neighborhoods walkability in the first quartile, the odds ratios of LTA for participants in the second, third and fourth quartile were 1.10 (95 % CI: 1.08–1.40), 1.23 (95 % CI: 1.09–1.56) and 1.42 (95 % CI: 1.11–1.82), respectively. Compared to participants with the first quartile of social participation, the odds ratios of LTA for participants in the second, third and fourth quartile of social participation were 2.02 (95 % CI: 1.57–2.60), 2.56 (95 % CI: 1. 98–3.31) and 4.69 (95 % CI: 3.63–6.06) respectively. However, **model 2** indicated there was only neighborhood-level walkability was positively associated with the prevalence of LTA without controlling for individual covariates. Compared to participants living in neighborhoods with the first quartile of walkability, the odds ratios of LTA for participants living in neighborhoods with the second, third and fourth quartile of walkability were 1.13 (95 % CI: 1.06–2.04), 1.76 (95 % CI: 1.09–3.24) and 1.83 (95 % CI: 1.10–3.72) respectively.

**Table 3:** The odds ratios and 95 % confidence intervals for LTA associated individual and neighborhood-level variables

	Model 1	Model 2	Model 3^a^
	OR (95 % CI)	OR (95 % CI)	OR (95 % CI)
Fixed effects			
Individual level variables			
Social cohesion			
1^st^ quartile	1		1
2^nd^ quartile	1.08 (1.00–1.14)		1.09 (1.07–1.20)
3^rd^ quartile	1.10 (1.04–1.44)		1.14 (1.08–1.50)
4^th^ quartile	1.28 (1.07–1.44)		1.31 (1.11–1.58)
Social participation			
1^st^ quartile	1		1
2^nd^ quartile	2.02 (1.57–2.60)		1.86 (1.44–2.41)
3^rd^ quartile	2.56 (1.98–3.31)		2.37 (1.82–3.08)
4^th^ quartile	4.69 (3.63–6.06)		4.27 (3.27–5.58)
Aesthetic quality			
1^st^ quartile	1		1
2^nd^ quartile	1.31 (0.90–1.68)		1.29 (0.89–1.66)
3^rd^ quartile	1.27 (0.99–1.62)		1.25 (0.97–1.60)
4^th^ quartile	1.23 (0.95–1.59)		1.14 (0.88–1.48)
Walkability			
1^st^ quartile	1		1
2^nd^ quartile	1.10 (1.08–1.40)		1.18 (1.10–1.51)
3^rd^ quartile	1.23 (1.09–1.56)		1.24 (1.09–1.57)
4^th^ quartile	1.42 (1.11–1.82)		1.41 (1.09–1.81)
Neighborhood level variables			
Social cohesion			
1^st^ quartile		1	1
2^nd^ quartile		0.81 (0.44–1.51)	0.79 (0.42–1.48)
3^rd^ quartile		1.20 (0.67–2.16)	1.27 (0.70–2.31)
4^th^ quartile		0.68 (0.35–1.31)	0.71 (0.36–1.40)
Social participation			
1^st^ quartile		1	1
2^nd^ quartile		0.93 (0.53–1.63)	0.82 (0.47–1.45)
3^rd^ quartile		1.18 (0.68–2.03)	0.97 (0.56–1.68)
4^th^ quartile		1.61 (0.92–2.80)	1.08 (0.61–1.90)
Aesthetic quality			
1^st^ quartile		1	1
2^nd^ quartile		0.86 (0.45–1.66)	0.79 (0.41–1.54)
3^rd^ quartile		0.84 (0.47–1.49)	0.96 (0.54–1.71)
4^th^ quartile		1.82 (0.97–3.41)	1.69 (0.89–3.23)
Walkability			
1^st^ quartile		1	1
2^nd^ quartile		1.14 (1.06–2.04)	1.13 (1.03–2.02)
3^rd^ quartile		1.76 (1.09–3.24)	1.73 (1.12–3.21)
4^th^ quartile		1.83 (1.10–3.27)	1.85 (1.19–3.35)
Random effects			
Neighborhood-level variance (SE)	0.39 (0.11)	0.32 (0.10)	0.32 (0.10)
Model fit			
-2LL	3506.96	3666.26	3438.85
AIC	3534.96	3694.26	3510.85

*LTA* leisure-time active, -*2LL* -2 log likelihood (smaller is better), *AIC* Akaike information criterion (smaller is better)

^a^Gender, Age, marital status, educational attainment, self-reported chronic diseases and self-rated health were adjusted

In **model 3**, individual- and neighborhood-level attributes of neighborhood were simultaneously entered into the model with controlling for individual covariates.. After controlling for individual covariates, individual-level social cohesion and social participation were still positively associated with the prevalence of LTA; meanwhile individual-perceived walkability and neighborhood-level walkability were still positively associated with the prevalence of LTA.

## Discussion

The present study examined the relationship between social and physical attributes of neighborhood with LTPA among elders by multilevel analysis methods in Mainland China. One of our findings indicated that only individual-level social participation were associated with LTPA, which was consistent with previous studies [29, 62] among whole population. Social participation measures the individual’s participation in several social activities within the life of modern society. There were several possible explanations why individual-level social participation was found to be associated with LTPA. Firstly, social participation may involve in participation of clubs or associations of recreational, physical and cultural activities. Secondly, social participation may increase one’s access to information about physical activity opportunities or the importance of physical activity for health [62]. Social cohesion is another aspect of the social environment of a neighborhood that has the potential to influence individual health and health-related behaviors such as physical activity [63]. Social cohesion refers to two inter-related features of society: (1) the absence of latent social conflict; and (2) the presence of strong social bonds-often measured by levels of trust and norms of reciprocity [31]. Cohesive communities may be better to reinforce positive social norms for health behaviors (e.g., physical activity) and lead to quicker or more widespread adoption of healthy behaviors because neighbors know and trust each other [25, 63]. In additional, neighbors that trust one another are more likely to provide helps and supports promoting access to services and amenities in time of need. Previous study among whole population in Sweden [64] has shown that low trust was positively associated with low LTPA. Another study among middle-aged and older adults in Australia [65] has shown that social cohesion was positively associated with LTPA. The current study indicated that individual-level social cohesion was also associated with LTPA among Chinese elderly.

Chinese have been proven to be more collectivistic [66], but social capital in China resides largely in families or in other narrow circles of social relationships. It implies that people may only trust those who belong to the same in-group and may not participate social activities outside of their circles [67]. When individual-level social participation and social cohesion were aggregated up to the neighborhood level, its effect on LTPA may tend to become diluted and less relevant. So there were no associations between neighborhood-level social participation and social cohesion with LTPA.

The current study examined the associations of two domains of physical neighborhood attributes, aesthetic quality and walkability with LTPA. Firstly, we found that there was no association between aesthetic quality and LTPA, which was consistent with previous studies among middle-aged adults in Shanghai [44]. Another study among whole population in Shanghai also shown that aesthetic quality wasn’t associated with leisure-time walking [48]. However, a previous study [46] in Hangzhou found that aesthetic quality was positively associated with LTPA and LTW (both measured as MET-min) among adult women, but not among adult men. Another study [47] among the elderly in Hong Kong showed that building attractiveness was positively associated with LWT, but not with LTPA other than walking. These contradictory findings suggest the overall aesthetic quality of a city may be important to LTPA, and a multicenter study including various cities may be needed to unpick these differences.

Walkable neighborhoods characterized by density, land used diversity, and well-connected transportation networks have been linked to more walking, less obesity, and lower coronary heart disease risk [68–70]. We found that both individual-level and neighborhood-level walkability of neighborhood were positively associated with LTPA, which were consistent with previous study [65]. However, a study among middle-aged adults in Shanghai indicated street connectivity was negatively associated with LTPA. One reason to explain these differences could be that the Chinese elderly are engaged in more LTPA than the Chinese youth [71]. These findings suggest that building walkable neighborhoods may promote LTPA among the elderly.

There are some limitations to our study. First, the direction of causality could not be addressed due to the cross-sectional study design. Second, even though IPAQ was positively associated with accelerometer-assessed physical activity [72], IPAQ often overestimates physical activity levels. Therefore the true number of individuals exercising >150 MET-min/week in this study population is likely an over-estimate. Third, because physical activity data were collected during the hottest months of summer (between July and September) rather than collected strategically across four seasons, so seasonal effects on physical activity should be noticed. Finally, a large sample from 47 neighborhoods were involved, but the study was conducted in only one administrative district of Shanghai, which may not be representative of the total elderly population in China. Multicenter well-designed prospective studies of neighborhood correlates of physical activity are warranted in the future.

## Conclusions

In spite of the above limitations, this study indicates that both social and physical attribute of neighborhood are associated with LTPA among the Chinese elderly. It may promote LTPA in Chinese elders to encourage them to participate in social activities, meanwhile, building walkable and cohesive neighborhoods.
